# Preparation of magnetite nanoparticles and their application in the removal of methylene blue dye from wastewater

**DOI:** 10.1038/s41598-024-69790-w

**Published:** 2024-08-29

**Authors:** Sohair T. Aly, Amgad Saed, Alaa Mahmoud, Mahmoud Badr, Shady S. Garas, Shehab Yahya, Kareem H. Hamad

**Affiliations:** Chemical Engineering Department, Egyptian Academy for Engineering and Advanced Technology, Cairo, 3056 Egypt

**Keywords:** Advanced oxidation method, Dye removal, Nanoparticles, Wastewater treatment, Environmental sciences, Chemistry, Engineering, Nanoscience and technology

## Abstract

Wastewater is discharged in large amounts from different industries; thus, wastewater treatment is currently one of the main concerns, advanced oxidation is a promising technique for wastewater treatment. This research aims to synthesize magnetite nanoparticles and study their application in wastewater treatment via adsorption and advanced oxidation processes. Magnetite nanoparticles were synthesized via coprecipitation technique between ferric and ferrous sulfate at a molar ratio of 2:1. The prepared sample was characterized using FTIR, XRD, TEM, BET surface area, zeta potential, VSM, and UV‒visible spectroscopy. XRD confirmed the formation of a single face-centered cubic (FCC) spinel structure of Fe_3_O_4_. TEM revealed an average particle size of 29.2 nm and a BET surface area of 70.1 m^2^ g^−1^. UV‒visible spectroscopy revealed that the UV–visible peak of the sample was obtained at 410 nm. VSM confirmed the attraction of the sample to a magnet with a magnetization of 60 (emu/g). The removal efficiency of methylene blue was studied using adsorption and advanced oxidation methods. For adsorption, the studied parameters were dye concentration 2–10 ppm, 3–10 pH, and 50:300 mg Fe_3_O_4_/L. For advanced oxidation, peroxide was used with nanomagnetite as a catalyst, and the studied parameters were pH 2–11, magnetite dose 20–200 PPM, and peroxide dose 500–2000 PPM. The removal efficiency by adsorption reached 95.11% by adding 50 mg of Fe_3_O_4_/L and 10 ppm dye conc at 6.5 pH; on the other hand, in advanced oxidation, it reached 98.5% by adding 110 PPM magnetite and 2000 ppm H_2_O_2_ at pH 11. The magnetite nanoparticles were reused for ten cycles of advanced oxidation, for a 10% reduction in removal efficiency at the tenth cycle.

## Introduction

One of the main challenges facing most countries is the gap between water resources and water needs due to several factors, such as population growth and industrial activities. As a result of industrialization, a large amount of water is consumed, and a large amount of wastewater is produced, which poses a great threat to the environment^1^.

Environmental concerns related to the manufacture and use of dyes have greatly increased over the past years, and dyes are unquestionably one of the main factors influencing the textile dye industry today, which consumes huge amount of dye^2^.

The discharge of dyes resulting from textile manufacturing, paper making, and leather dyeing into water drains, canals, and rivers is considered the biggest problem facing the environment and threatening the safety of water and marine life.

Traditional treatment methods for effluent wastewater are physical, chemical, and biological methods. Adsorption transfers pollutants found in the liquid phase to the solid phase, after which further processing is needed to address the contaminated sludge. This is considered the main problem in all traditional methods^3–5^.

One of the most important techniques used in wastewater treatment is the advanced oxidation process (AOP). Heterogeneous photocatalysis is an AOP that has several advantages, including the ability to mineralize pollutants, low cost, waste-free nature, and eco-friendliness^6–8^. The mechanism of action of AOPs mainly depends on the generation of highly reactive free radicals such as hydroxyl radicals (·OH), sulfate radicals (SO_4_·−), and superoxide radicals (O_2_·−) by the activation of different oxidants (hydrogen peroxide, persulfate/peroxydisulfate, peroxymonosulfate and sodium percarbonate via oxidation and reduction reactions^9–12^. These released radicals cause the degradation of organic pollutants and subsequent mineralization to inorganic ions, water and carbon dioxide^13,14^.

Iron-based materials are recommended for use as catalysts for the activation of heterogeneous AOPs due to their environmental friendliness and cost effectiveness, which make them suitable for large-scale practical applications^15,16^.

Magnetite-based catalysts are effective Fenton catalysts for generating HO· for the removal of organic pollutants in wastewater^17^. The magnetite catalyst has a wider working pH range. In the Fenton system, a series of reactions that mainly occur on the surface of minerals are shown in Eqs. (1–4) ^18^.

1 $$ {\text{Fe}}\left( {{\text{II}}} \right) + {\text{H}}_{{2}} {\text{O}}_{{2}} \to {\text{Fe}}\left( {{\text{III}}} \right) + {\text{OH}} - + {\text{ HO}} \cdot $$

2 $$ {\text{Fe}}\left( {{\text{III}}} \right) + {\text{H}}_{{2}} {\text{O}}_{{2}} \to {\text{Fe}}\left( {{\text{III}}} \right)\left( {{\text{H}}_{{2}} {\text{O}}_{{2}} } \right) $$

3 $$ {\text{Fe}}\left( {{\text{III}}} \right)\left( {{\text{H}}_{{2}} {\text{O2}}} \right) \to \left( {{\text{Fe}}\left( {{\text{II}}} \right) \cdot {\text{O}}_{{2}} {\text{H}}} \right) \, + {\text{ H}}_{{2}} {\text{O}} $$

4 $$ {\text{(Fe}}\left( {{\text{II}}} \right) \cdot {\text{O}}_{{2}} {\text{H}}) \to {\text{ Fe}}\left( {{\text{II}}} \right) \, + {\text{ HO}}_{{2}} \cdot /{\text{O}}^{{ \cdot - { 2}}} $$

The application of nanomagnetite as a catalyst in the AOP process is recommended because it has magnetic properties that result in high catalytic efficiency, and it can be separated easily from treated water^19–21^.

Response surface methodology (RSM) is a statistical software package that provides researchers with the ability to study the parameters affecting any process. On the other hand, process optimization can be performed using a predictive model based on a set of experiments that correlate the response to different variables^22–24^.

The response function (f) largely depends on the nature of the relationship between the response and the independent variables. The polynomial quadratic model is represented by Eq. (5):

5 $$ {\mathbf{Y}} = \upbeta_{0} \, + \,\upbeta_{{1}} \,{\text{X}}_{{1}} \, + \,\upbeta_{{2}} \,{\text{X}}_{{2}} \, + \,\upbeta_{{3}} \,{\text{X}}_{{3}} \, + \,\upbeta_{{4}} \,{\text{X}}_{{1}} \,{\text{X}}_{{2}} \, + \,\upbeta_{{5}} \,{\text{X}}_{{2}} \,{\text{X}}_{{3}} \, + \,\upbeta_{{6}} \,{\text{X}}_{{1}} \,{\text{X}}_{{3}} \, + \,\upbeta_{{7}} \,{\text{X}}_{{1}}^{{2}} \, + \,\upbeta_{{8}} \,{\text{X}}_{2}^{2} \, + \,\upbeta_{{9}} \,{\text{X}}_{3}^{2} $$

where Y is the intercept or regression coefficient; β_i_, β_ii,_ and β_ij_ represent the linear quadratic and interaction coefficients, respectively; and X_i_ and X_j_ are the coded values of the process variables^22^.

In this research, the RSM Box–Behnkin method was used to model and optimize the use of synthetic magnetite nanoparticles for both adsorption and advanced oxidation. Depending on the magnetic properties of the synthesized nanomagnetite, it was separated by a magnet to study its reuse in water treatment for several cycles, after which the removal efficiency was calculated.

## Experimental work

The raw material used, the method of preparation of nanomagnetite, the characterization methods, and the experimental work are explained.

### Raw materials

Sodium hydroxide (NaOH) (96% purity), ferric sulfate pentahydrate (Fe_2_(SO_4_)_3_·5H_2_O) (98% purity), and ferrous sulfate heptahydrate (FeSO_4_·7H_2_O) (98% purity) were purchased from Alpha Chemical.

### Preparation of magnetite nanoparticles

**Figure 1:**
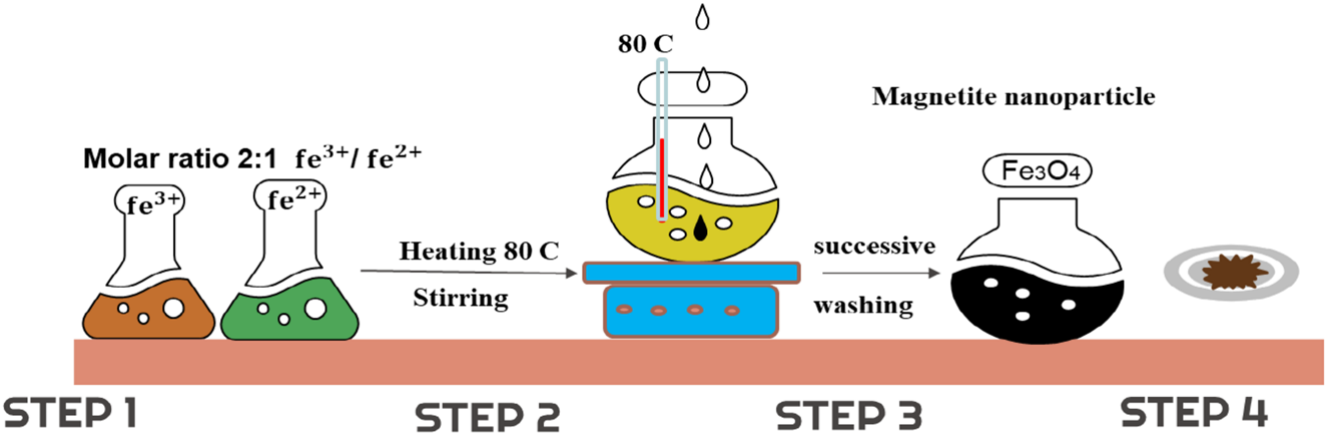
Graphical representation of the coprecipitation process for the synthesis of magnetite nanoparticles.

Magnetite nanoparticles (Fe_3_O_4_) were synthesized via the coprecipitation technique by mixing Fe_2_(SO_4_)_3_ and Fe(SO_4_) according to their stoichiometric ratios, as shown in Eq. (6), in distilled water. A solution of sodium hydroxide was added dropwise while stirring to reach a pH of 11. The mixture was continuously stirred and heated to 80 °C. Finally, the nanoparticles were separated, washed with distilled water to adjust the pH to 7.0, and then dried in an electrical dryer at 105 °C,^25^ as shown in Fig. 1.

6 $$ {\text{FeSO}}_{{4}} + {\text{Fe}}_{{2}} ({\text{SO}}_{{4}} )_{{3}} + {\text{ 8NaOH}} \to {\text{Fe}}_{{3}} {\text{O}}_{{4}} + {\text{ 4Na}}_{{2}} {\text{SO}}_{{4}} + {\text{4H}}_{{2}} {\text{O}} $$

### Characterization of magnetite nanoparticles

Characterization techniques were used to verify the properties of the magnetite nanoparticles via X-ray diffraction (XRD) using a Bruker D8 Advance ECO diffractometer in reflection mode. Fourier transform infrared spectroscopy, which was performed on an ATR-IR instrument (400–4000 cm^−1^), was carried out using an ALPHA II BRUKER, USA. Transmission electron microscopy (TEM) was also performed on an FEI Talos F200S field-emission transmission electron microscope with an accelerating voltage of 200 kV. The Brunauer–Emmett–Teller (BET) surface area was determined from a nitrogen adsorption isotherm at 77 K using a Belsorp adsorption automatic specific surface area analyzer (Microtrac BEL, Japan), vibrating sample magnetometer analysis (VSM), chemical oxygen demand (COD), zeta potential, and ultraviolet spectroscopy (UV‒VIS spectrophotometer) were used, as discussed below.

### Application of magnetite nanoparticles

The use of synthesized nanoparticles in adsorption and advanced oxidation was carried out. To study the adsorption of methylene blue using magnetite, three parameters were studied: dye concentration, 2:10 ppm; pH, 3:10 (effect of acidity and alkalinity of solution on removal efficiency); and magnetite dose in the range of 50:300 mg Fe_3_O_4_/L. RSM was used for the experiments, and both the adsorption isotherm and adsorption kinetics were studied. The concentrations of the different solutions were measured using a Spectro UV‒VIS Double-Model UVD-2950.

The oxidation process was performed using a sample of a synthetic solution with 100 ppm methylene blue dye (M. B.). The studied parameters were as follows: nanomagnetite dose, 20 to 200 ppm; peroxide dose, 500 to 2000 ppm; and pH 2:11 (from acidic to alkaline medium). The concentrations of the different solutions were measured using a Spectro UV‒VIS Double-Model UVD-2950. Table 4 shows the removal efficiency of dye from wastewater under different conditions. The removal efficiency was calculated as follows: (Ci − C_f_) × 100/C_i_, where Ci and C_f_ are the initial and final concentrations of dye, respectively. The highest removal efficiency (98.5%) was obtained by adding 110 mg of magnetite, 2000 ppm H_2_O_2,_ and a pH of 11.

## Results and discussion

The characterization of the prepared nanomagnetite and its application in wastewater treatment are discussed in the following sections.

### X-ray diffraction (XRD)

**Figure 2:**
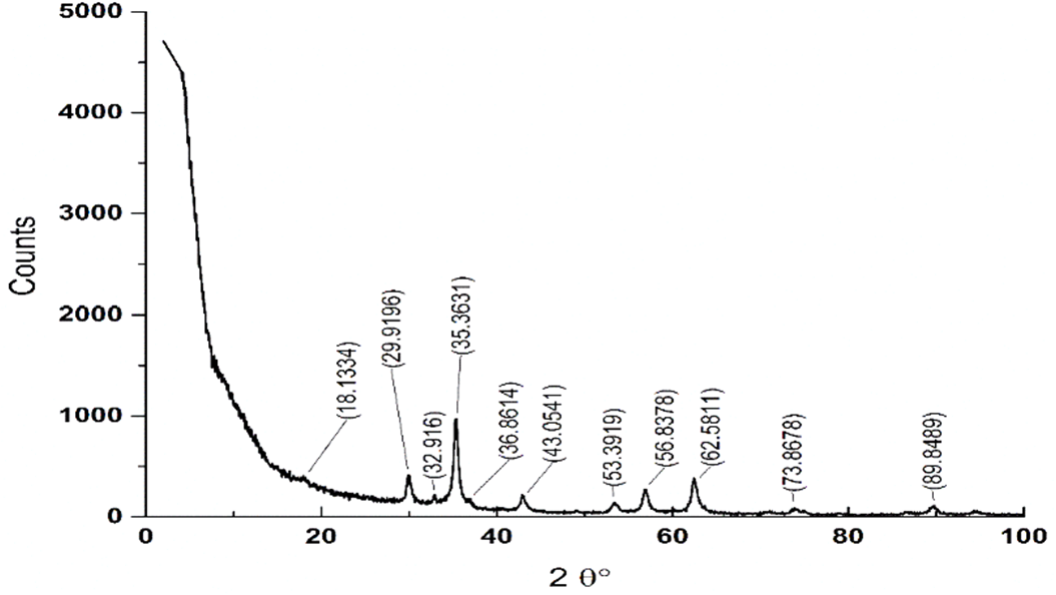
XRD of prepared magnetite-nanoparticles.

XRD was applied to determine the crystallinity of the tested sample. The peaks in the XRD pattern shown in Fig. 2 are between 30° and 62°, which correspond to standard magnetite^26^. These findings revealed that a single face-centered cubic (FCC) spinel structure of Fe_3_O_4_ was formed. (Device model SEIFERT XRD 3003 TT DIFRACTOR (GE, Germany) Equipped with a primary monochromator (CuK radiation, 2 ceta = 3 − 90°).

### Transmission electron microscopy (TEM)

**Figure 3:**
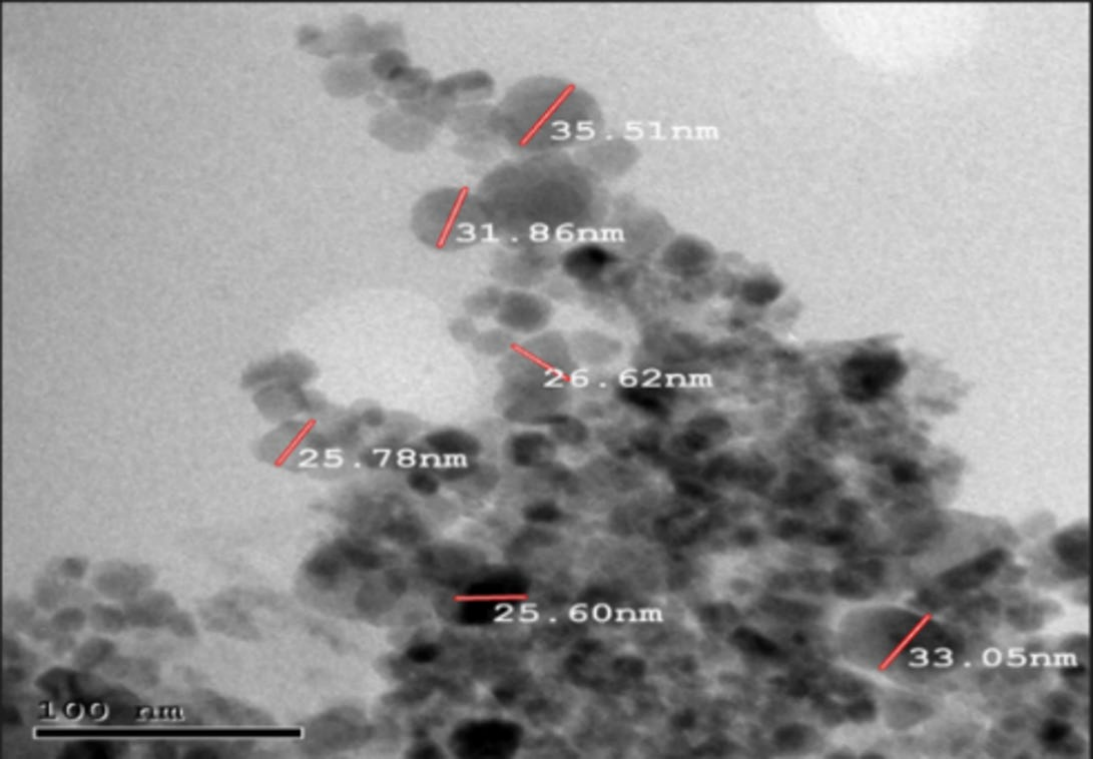
TEM image of the prepared magnetite nanoparticles.

TEM is essential for material science and has many characteristics, such as particle size and morphology. As shown in Fig. 3, the particles are almost spherical in shape, and the average particle size is 29.2 nm, which is a reasonable size for obtaining a high surface area. (Device model: JEOL JEM-2100 (Origin. Japan). The BET surface area is 70.1 m^2^ g^−1^.

### Zeta potential

**Figure 4:**
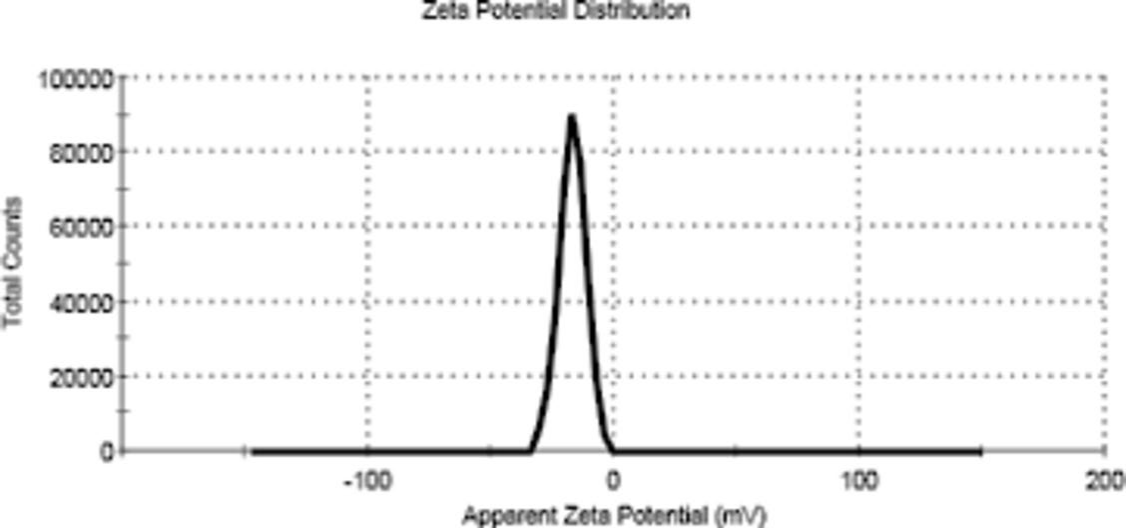
Zeta potential of the prepared magnetite nanoparticles.

The zeta potential is the charge that appears at the interface between a solid surface and the surrounding liquid medium. Figure 4 shows the results obtained from the examined sample, which indicate that the surface of magnetite has a negative charge when a particle is dispersed in a liquid, and the functional groups on its surface will react with the positively charged ions in the surrounding medium (device model: MALVERN ZETASIZER, USA).

### Fourier transform infrared (FTIR) spectroscopy

**Figure 5:**
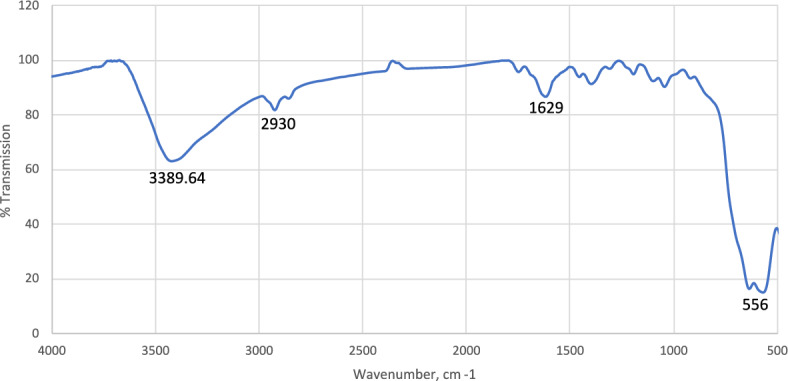
FTIR of prepared magnetite-nanoparticle.

The FTIR transmittance spectrum analysis is shown in Fig. 5. A peak is observed at 556 cm^−1^, which corresponds to the stretching vibration of the Fe–O bonds in the sublattice of Fe_3_O_4_. The peak at 2930 cm^−1^ is attributed to –CH_2_ and chemical group stretching vibrations. The values at 1629 cm^−1^ and 3389.64 cm^−1^ indicate the stretching of C–O, C=C, and OH–, respectively^27^. (Device model (FTIR) JASCO, FTIR-300 E.

### UV–visible spectroscopy

**Figure 6:**
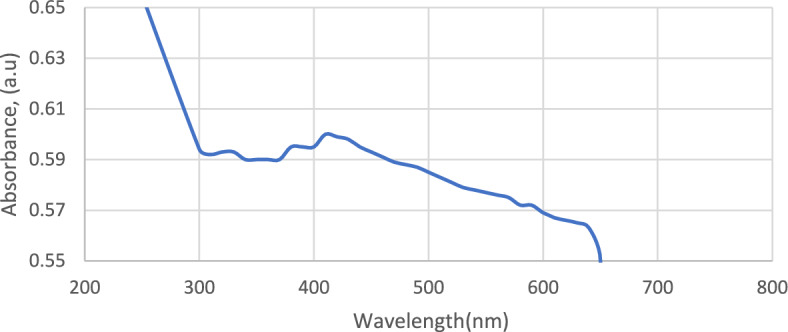
UV–visible absorption spectroscopic analysis of the prepared magnetite nanoparticles indicating a peak at 410 nm.

The UV–visible spectra of the prepared magnetite nanoparticles are displayed in Fig. 6. The UV–visible peak of the sample was obtained at 410 nm. The reported UV–visible peak was found to be at 407 nm by Suresh Kumar et al.^28^. The peak in the near-IR region confirmed the presence of magnetite nanoparticles.

### VSM of synthetic nanomagnetite

**Figure 7:**
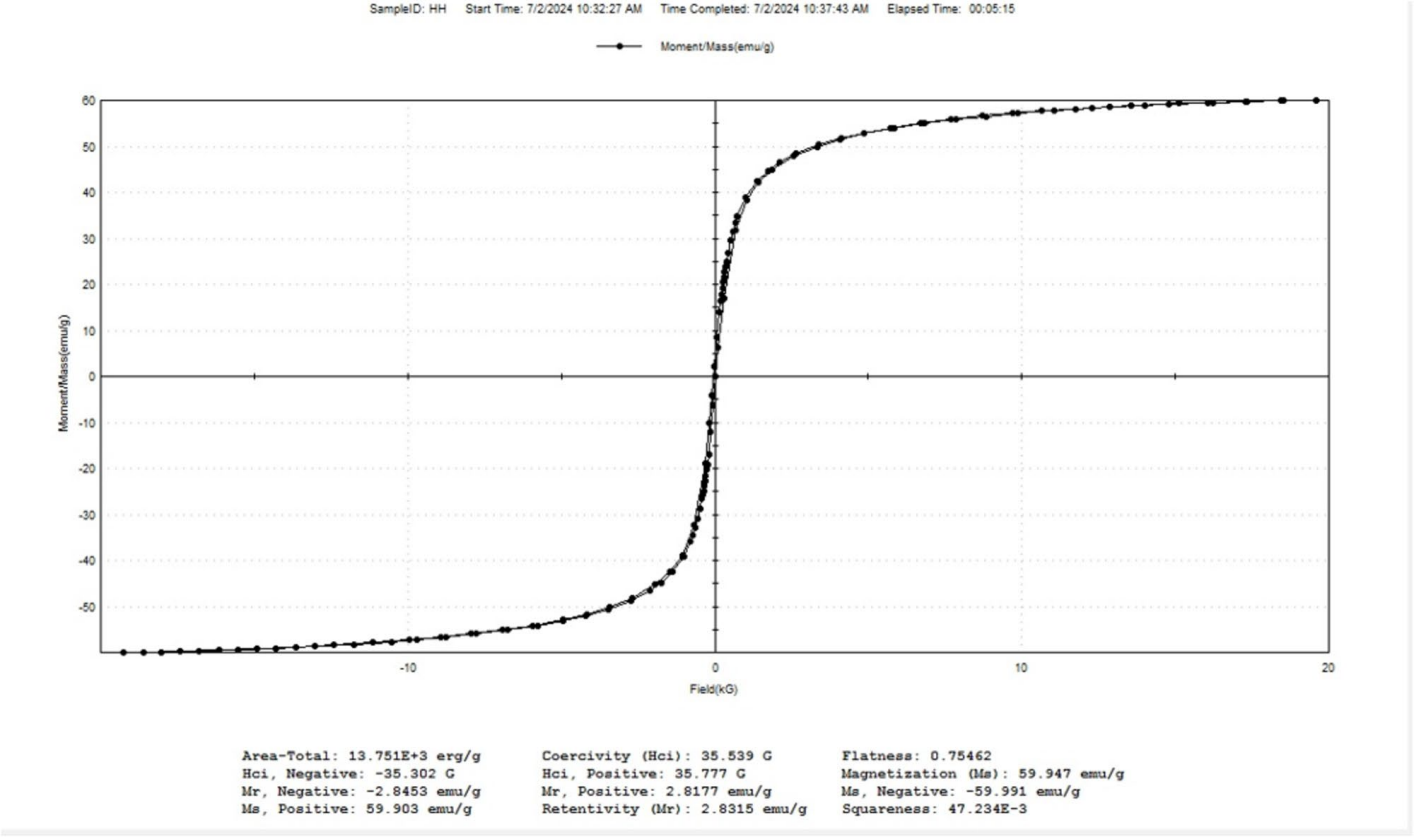
VSM of synthetic nanomagnetite.

Vibrating sample magnetometer analysis (VSM) was performed using a Lake shore model 7410 instrument to analyze the synthesized nanomagnetite, as shown in Fig. 7. As shown in the figure, the magnetization of the sample revealed that it was completely attracted to the magnet and had a magnetization of 60 (emu/g)^29^.

**Figure 8:**
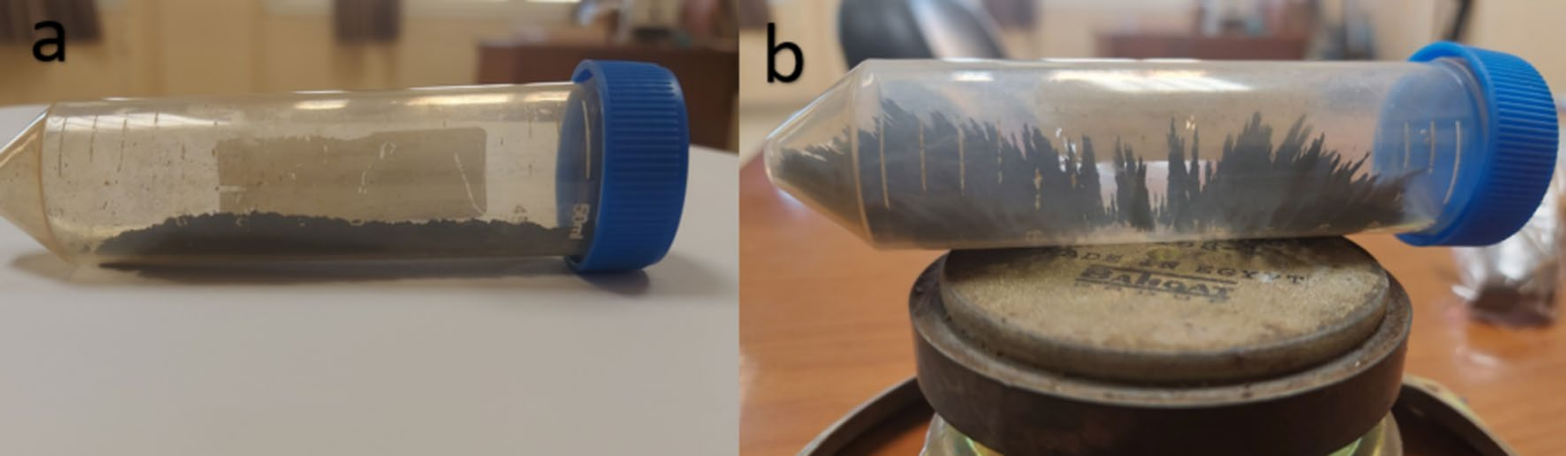
Produced nanomagnetite (**a**) before being subjected to a magnetic field (**b**) after being subjected to a magnetic field.

Figure 8 shows the pattern taken by the nanoparticles when subjected to a magnetic field, which confirms its magnetic properties.

### Adsorption of methylene blue dye using magnetite nanoparticles

The results obtained in different runs are tabulated in Table 1. The highest removal efficiency (95.11%) was obtained by adding 50 mg of Fe_3_O_4_/L, 10 ppm dye conc and 6.5 pH.

**Table 1:** Removal efficiency of methylene blue dye using nanomagnetite under different conditions.

Run	A: pH	B, concentration ppm	C: dose, mg	Removal efficiency%
1	10	10	175	85.2
2	3	6	50	37.1
3	10	2	175	32.6
4	3	6	300	31.8
5	3	2	175	79.8
6	6.5	6	175	77.2
7	6.5	6	175	82.2
8	6.5	2	50	15
9	6.5	10	50	95.1
10	6.5	10	300	77.98
11	3	10	175	81.4
12	6.5	6	175	82.2
13	10	6	50	84.4
14	10	6	300	53.3
15	6.5	2	300	14.8
16	6.5	6	175	85.1
17	6.5	6	175	77.6

The reduced cubic model was the best model for representing the obtained data, with an R^2^ equal to 0.973 and an adjusted R^2^ of 0.94. Table 2 displays an ANOVA of the obtained model, which indicates that the model is significant with a *p* value of 0.0001. The suggested model that relates the studied parameters and removal efficiency according to the obtained results and statistical analysis was a reduced cubic model with R^2^ = 0.9697, adjusted R^2^ = 0.9395, and a *p* value lower than 0.0001, which confirmed that the model was significant. The ANOVA results of the models are displayed in Table 6. These results indicate that the model is significant and has a high confidence level, as the *p* value is lower than 0.05 and the F value is 32, which reveals the importance of the variance in each variable^30^.

**Table 2:** ANOVA for the reduced cubic model.

Source	Sum of squares	df	Mean square	F value	*p* value	
Model	11,421.36	9	1269.04	28.04	0.0001	Significant
A-pH	472.41	1	472.41	10.44	0.0144	
B-concentration	5136.59	1	5136.59	113.51	< 0.0001	
C-dose	362.66	1	362.66	8.01	0.0254	
AB	648.47	1	648.47	14.33	0.0068	
AC	165.34	1	165.34	3.65	0.0976	
A^2^	124.33	1	124.33	2.75	0.1414	
C^2^	2520.74	1	2520.74	55.70	0.0001	
A^2^B	991.68	1	991.68	21.91	0.0023	
AC^2^	1576.04	1	1576.04	34.83	0.0006	

#### Dye removal equations by adsorption

The removal efficiency (Y) equation was obtained from the statistical analysis of the coded values

7 $$ \begin{aligned} {\text{Y}} = & {78}.{33}{-}{1}0.{\text{87 pH}} + {35}.{\text{84 Conc}}{-}{6}.{\text{73 dose}} + {12}.{\text{73 pH}}*{\text{Conc}}{-}{6}.{\text{43 pH}}*{\text{dose}} \\ & {-}\,{5}.{\text{43 pH}}^{{2}} {-}{24}.{\text{43 dose}}^{{2}} {-}{22}.{\text{27 pH}}^{{2}} *{\text{conc}} + {28}.0{\text{7 pH}}*{\text{dose}}^{{2}} \\ \end{aligned} $$

The removal efficiency (Y) equation was obtained from the statistical analysis of the actual values

8 $$ \begin{aligned} {\text{Y}} = & {19}.{34}{-}{19}.{\text{957 pH}}{-}{16}.{\text{15 Conc}} + {1}.{\text{756 Dose}} + {6}.{\text{81 pH}}*{\text{Conc}} \\ & {-}\,0.{\text{194 pH}}*{\text{dose}} + {2}.{\text{283 pH}}^{{2}} {-}0.000{\text{49 dose}}^{{2}} {-}0.{\text{454 pH}}^{{2}} *{\text{Conc}} \\ & + \,0.000{\text{513 pH}}*{\text{dose}}^{{2}} \\ \end{aligned} $$

#### Interaction between the studied parameters

A 2-D plot can be drawn for different variations in parameters, which exhibit a trend in which the response varies within the selected range of input parameters and the effect of each parameter over the other parameters. With the aid of statistical analysis, the interactions between the three studied parameters, namely, pH “A”, dye concentration “B” and dose “C”, can be studied using the obtained model graph contours.

**Figure 9:**
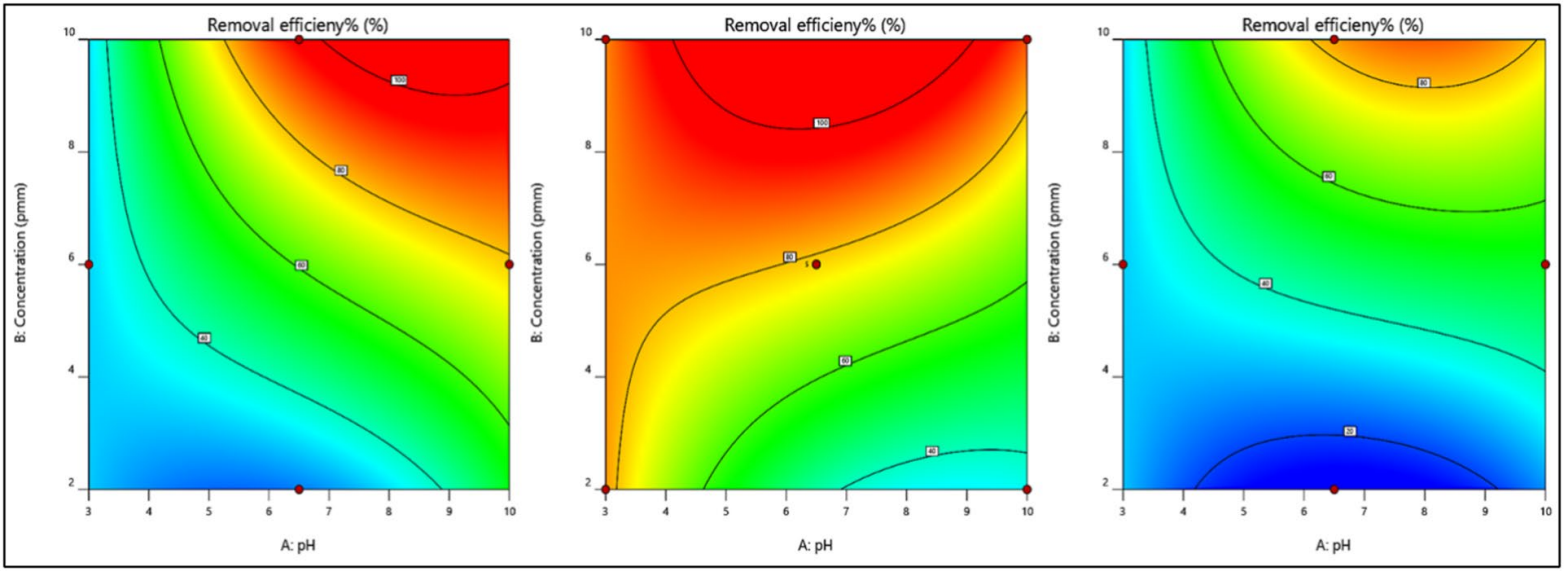
Effect of dye concentration and pH on dye removal for nanomagnetite at (**a**) minimum, (**b**) medium, and (**c**) maximum concentrations.
- Effect of the magnetite dose and pH on dye removal
  The effect of magnetite dose and pH on dye removal efficiency is illustrated in Fig. 10a–c. As the concentration of dye increases from the minimum to the maximum value, the removal efficiency increases even at low pH and magnetite doses.

- Effect of dye concentration and pH on dye removal
  The effects of pH and concentration on removal efficiency are displayed in Fig. 9a–c. At a low magnetite dose, the removal efficiency increases as the pH and concentration increase simultaneously. At an average dose of magnetite, increasing the concentration of dye increases the removal efficiency even at a low pH. At a high dose of magnetite, the removal efficiency increases with increasing pH and concentration simultaneously, which confirms the interaction between the studied parameters.

**Figure 10:**
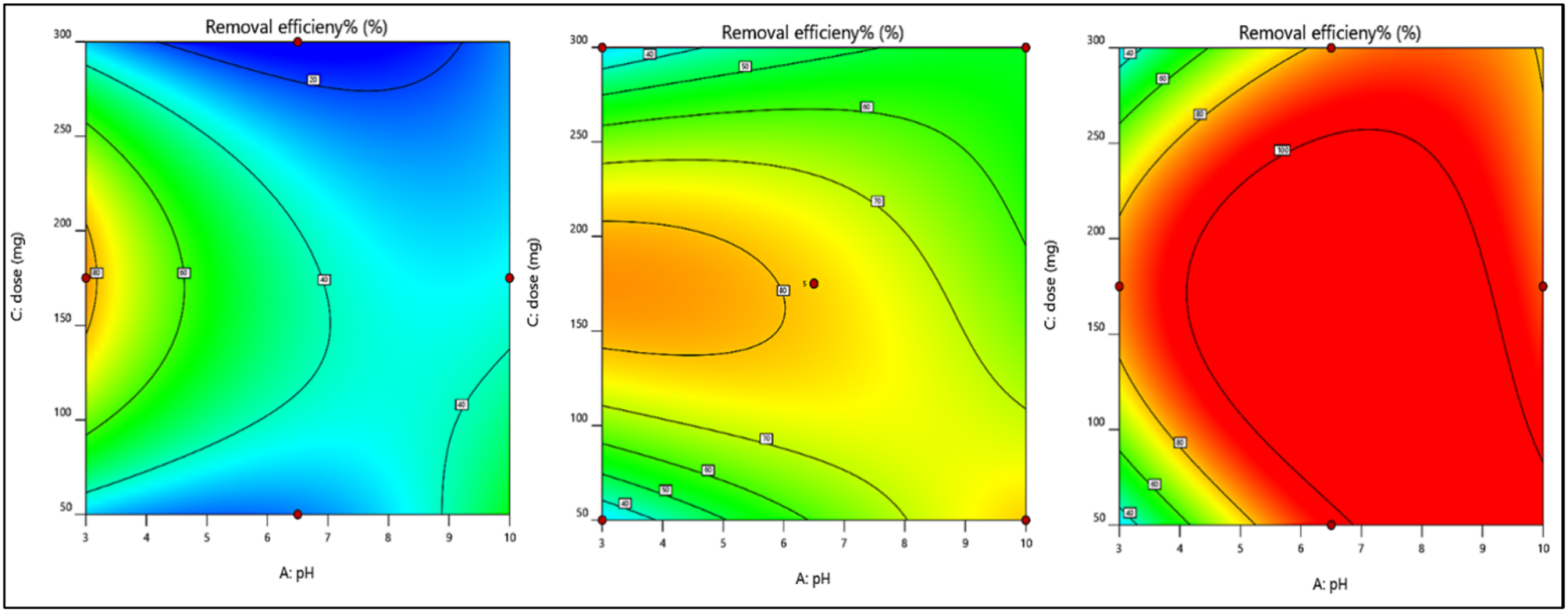
Effect of pH and nanomagnetite concentration on the removal efficiency of Dye Conc. at (**a**) minimum, (**b**) medium, and (**c**) maximum values.

#### Adsorption isotherm

Synthetic wastewater solutions with concentrations of 10, 20, 40, 60, 80, and 100 ppm methylene blue dye were prepared. The dose of magnetite was 0.05 g/L for all the samples. All the samples were agitated on a mechanical shaker at a speed equal to 100 rpm. The concentrations of all the samples were measured over time until they reached equilibrium. The results are listed in Table 3.

**Table 3:** Equilibrium concentrations for different samples.

Ci, ppm	10	20	40	60	80	100
Ce, ppm	0.5	1.9	22.2	36.1	43.2	77.2
qe, mg/gm	19	36.2	35.6	47.8	73.6	45.6

The adsorption capacity increased with increasing concentration up to 80 ppm and then decreased at 100 ppm, possibly due to the saturation of magnetite.

Three models were applied: the Langmuir isotherm model, Freundlich isotherm model, and Dubinin–Radushkevich isotherm model.

**Figure 11:**
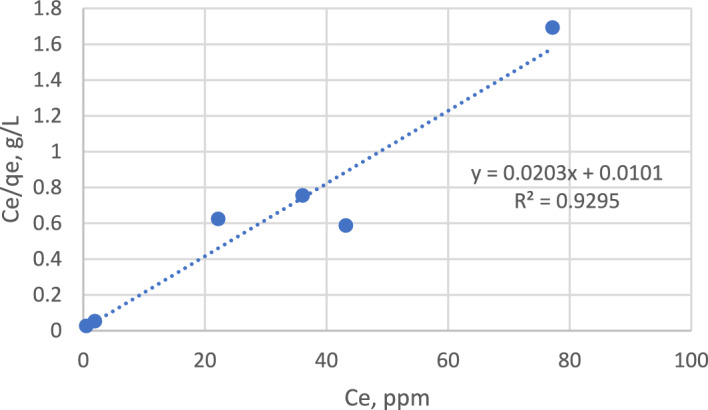
Langmuir isotherm model.

**Figure 12:**
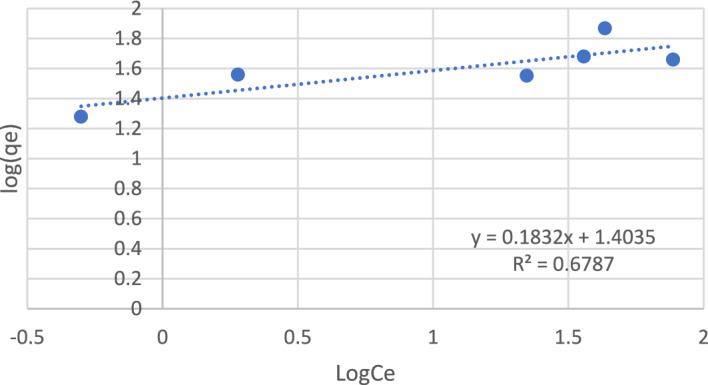
Freundlich isotherm model.

As shown in Figs. 11 and 12, the Langmuir isotherm was fitted with the experimental data, as it had an R^2^ of 0.9295; in contrast, the Freundlich isotherm had an R^2^ of 0.6787.

The maximum adsorption capacity was calculated from the line slope, which was q_max_ = 1/slope = 50 mg/g, and the Langmuir constant KL = 2 L/mg.

The Langmuir model indicates that a monolayer is formed, the heat of adsorption Q is constant and independent of coverage, each adsorbate molecule occupies only one site, and the adsorption is localized (molecules remain at the site of adsorption until desorption).

The Dubinin–Radushkevich isotherm model (D–R) can be applied to determine whether the adsorption process is physical or chemical and is expressed as^31^

9 $$ {\text{ln qe}} = {\text{ln K}}_{{{\text{D}} - {\text{R}}}} - \upbeta \upvarepsilon^{{2}} $$

where K_D−R_ (mg g^−1^) is the Dubinin–Radushkevich constant and the Polanyi potential is ε (mol^2^ J^−2^), which is equal to

10 $$ \varepsilon = {\text{RT}}\,{\text{ln}}\left( {{1} + {1}/{\text{Ce}}} \right) $$

where T is the absolute temperature (K) and R is the universal gas constant (8.314 J K^−1^ mol^−1^). The constant β is related to E (kJ mol^−1^). The energy E is defined as the free energy change required to transfer 1 mol of ions from the solution to the solid:

11 $$ E = \frac{1}{{sqrt\left( {2B} \right)}} $$

**Figure 13:**
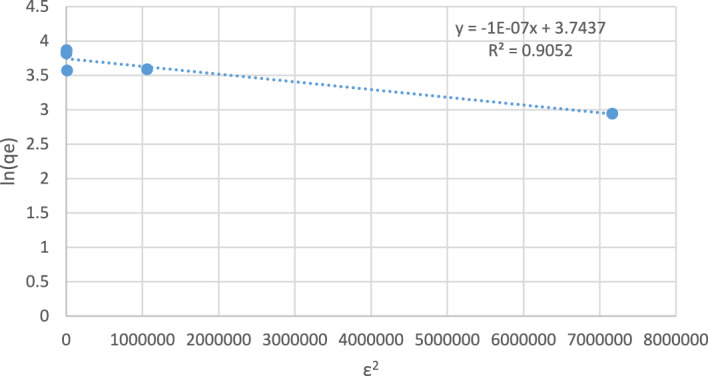
Dubinin–Radushkevich isotherm.

The linear relation of (ln qe) against ε^2^ is carried out as shown in Fig. 13, and the values β and K_D−R_ are obtained from the slope and intercept of the line. The value of E represents the information adsorption mechanism; an E value less than 8 kJ mol^−1^ represents the physisorption process, and an E value within the range of 8–16 kJ mol^−1^ is assigned to the chemisorption process^31^. The calculated value of E is 2.23 kJ/mol, which indicates that the adsorption of M.B by the nanomagnetite physisorption process.

#### Adsorption kinetics

**Figure 14:**
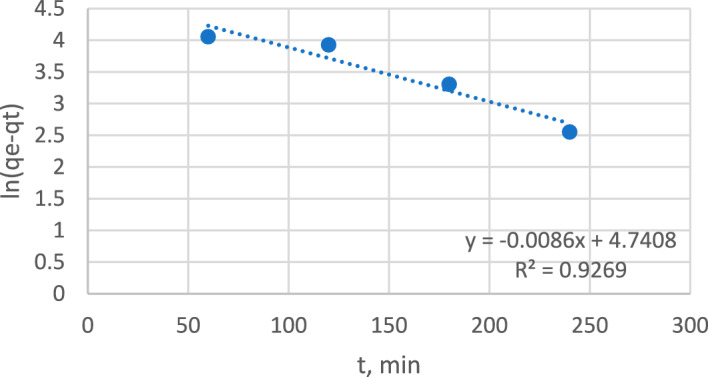
Pseudo-first-order model.

**Figure 15:**
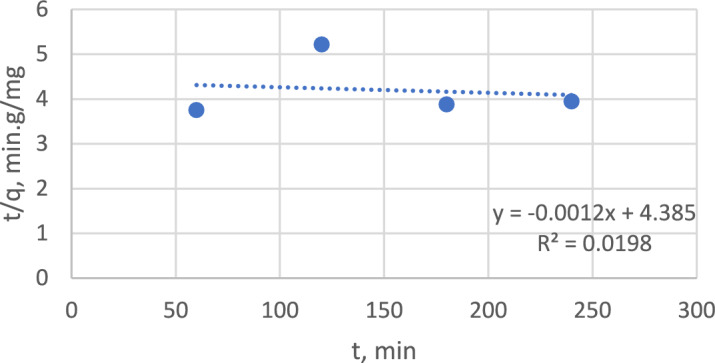
Pseudo-second order model.

Adsorption kinetics were studied for a sample with an 80 ppm concentration, as it maintained its maximum adsorption capacity. Two kinetic models were applied: a pseudo-first-order model and a pseudo-second-order model. Figures 14 and 15 show that the pseudo first-order model fit the experimental data, as it had an R^2^ of 0.9269 versus an R^2^ of 0.0198 for the pseudo second-order model.

#### Thermodynamic study

**Figure 16:**
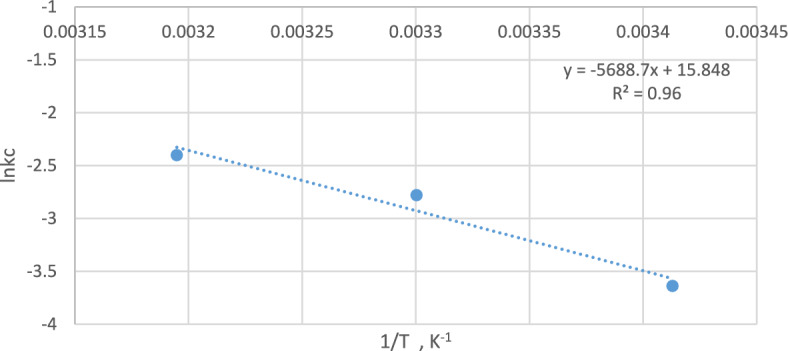
Linear plot of thermodynamic parameters for the adsorption of M.B.

A thermodynamic study was carried out by changing the temperature (20, 30, 40 °C) during the adsorption of methylene blue dye with nanomagnetite. Determination of the equilibrium concentration of different samples and thermodynamic parameters such as the free energy change (ΔG^0^), enthalpy change (ΔH^0^) and entropy change (ΔS^0^) were carried out using the following equations^32^:

12 $$ \Delta {\text{G}}^{0} = \Delta {\text{H}}^{0} - {\text{T}}\Delta {\text{S}}^{0} $$

13 $$ \Delta {\text{G}}^{0} = - \,{\text{RT}}\,{\text{ln}}\left( {\text{k}} \right){\text{c}} $$

where T is the temperature in K, R is the universal gas constant (R = 8.314 J mol^–1^ K^–1^), and Kc is the equilibrium constant (kc = q_e_/C_e_). A linear plot between ln (Kc) and 1/T is displayed in Fig. 16. The values of ΔS^0^ and ΔH^0^ were determined from the intercept and slope, respectively, as presented in Table 4. The value of ΔH^0^ is positive, which points to an endothermic reaction. The value of ΔS^0^ is positive, which indicates that the degrees of freedom increased at the solid–liquid interface during dye adsorption.

**Table 4:** Thermodynamic parameter values for the adsorption of M.B dye onto nanomagnetite.

Temperature, K	ln kc	ΔG^0^(kJ mol^−1^)	ΔH^0^(kJ mol^−1^)	ΔS^0^(J mol^−1^ K^−1^)	R^2^
293	− 3.6381	17.83557	56.4	131.76	0.96
303	− 2.781	16.51797			
313	− 2.402	15.20037			

### Application of advanced oxidation in removing methylene blue dye using nanomagnetite as a catalyst

The parameters and removal efficiency are tabulated in Table 5. The suggested model that relates the studied parameters and removal efficiency according to the obtained results and statistical analysis was a reduced cubic model with R^2^ = 0.9697, adjusted R^2^ = 0.9395, and a *p* value lower than 0.0001, which confirmed that the model was significant. The ANOVA results of the models are displayed in Table 6. These results indicate that the model is significant and has a high confidence level, as the *p* value is lower than 0.05 and the F value is 32, which reveals the importance of the variance in each variable^30^. The maximum removal percentage reached 98.5% according to the tabulated values; this value is greater than that of most of the methods found in the literature^33^, and not only does this removal occur because the AOP does not produce any sludge.

**Table 5:** Removal efficiency of methylene blue dye using magnetite and H2O2 under different conditions.

Run	A: Cata. Dose, mg	B: peroxide dose, PPM	C: pH	%removal efficiency (C_i_ − C_f_) × 100/C_i_
1	110	2000	11	98.5
2	110	1250	6.5	0
3	110	1250	6.5	0
4	200	500	6.5	10.5
5	110	1250	6.5	0
6	110	2000	2	53.3
7	200	1250	11	96.3
8	110	1250	6.5	0
9	110	1250	6.5	0
10	20	2000	6.5	84.7
11	110	500	2	47.42
12	20	1250	11	96.7
13	20	500	6.5	46.3
14	200	2000	6.5	86.1
15	110	500	11	79.7
16	200	1250	2	92.1
17	20	1250	2	72.7

**Table 6:** ANOVA for the reduced cubic model.

Source	Sum of squares	df	Mean square	F value	*p* value	
Model	25,633.59	8	3204.20	32.05	< 0.0001	Significant
A-Cata.Dose	29.65	1	29.65	0.2965	0.6009	
B-peroxide dose	152.28	1	152.28	1.52	0.2522	
C-pH	1396.03	1	1396.03	13.96	0.0057	
AB	345.96	1	345.96	3.46	0.0999	
A^2^	6179.60	1	6179.60	61.82	< 0.0001	
B^2^	1455.11	1	1455.11	14.56	0.0051	
C^2^	11,011.79	1	11,011.79	110.15	< 0.0001	
A^2^B	997.26	1	997.26	9.98	0.0134	

#### Dye removal efficiency equations using the advanced oxidation approach

The removal efficiency (Y) equation obtained from the statistical analysis of the coded values is shown below:

14 $$ \begin{aligned} {\text{Y}} = & 0{-}{1}.{\text{92 dose}} + {6}.{\text{17 Hydrogen peroxide}} + 0{13}.{\text{12 PH}} \\ & + \,{9}.{3}0{\text{ dose}}*{\text{Hydrogen peroxide}} + {38}.{\text{31 dose}}^{2} \\ & + \,{18}.{\text{59 Hydrogen peroxide}}^{2} + {51}.{\text{14PH}}^{2} \\ & + \,{22}.{\text{33 dose}}^{2} *{\text{Hydrogen peroxide}} \\ \end{aligned} $$

The actual removal efficiency is as follows:

15 $$ \begin{aligned} {\text{Y}} = & {151}.{9}{-}0.{\text{223 dose}}{-}0.0{\text{45 Hydrogen peroxide}}{-}{29}.{\text{89 dose}} \\ & {-}\,0.000{\text{671 Hydrogen peroxide}} + 0.000{\text{135 dose}}^{{2}} \\ & + \,0.0000{\text{33 Hydrogen peroxide}}^{{2}} + {2}.{\text{525pH}}^{{2}} \\ & + \,{3}.{\text{675 dose}}^{{2}} *{\text{ Hydrogen peroxide}} \\ \end{aligned} $$

#### Effect of the catalyst dose and hydrogen peroxide on dye removal (interaction between study parameters)

**Figure 17:**
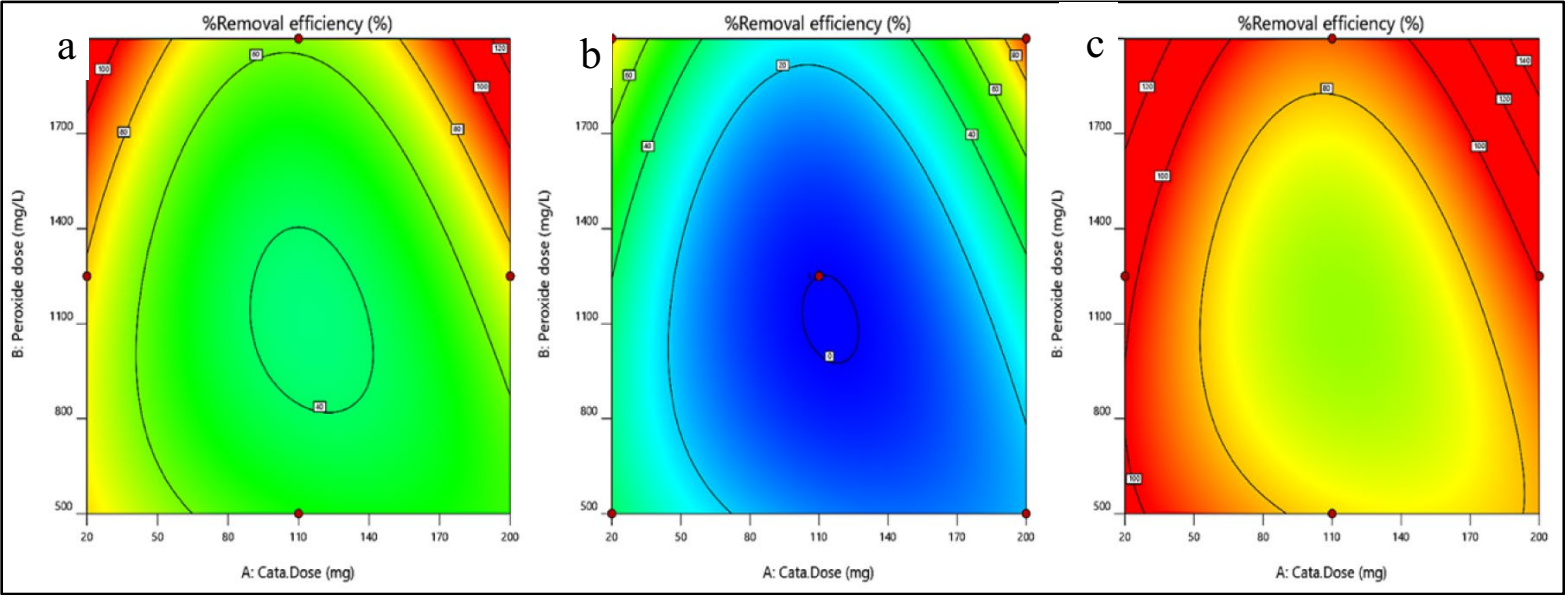
Effect of the catalytic dose and hydrogen peroxide on the COD with respect to the pH at the (**a**) Minimum, (**b**) Medium, and (**c**) Maximum values.

**Figure 18:**
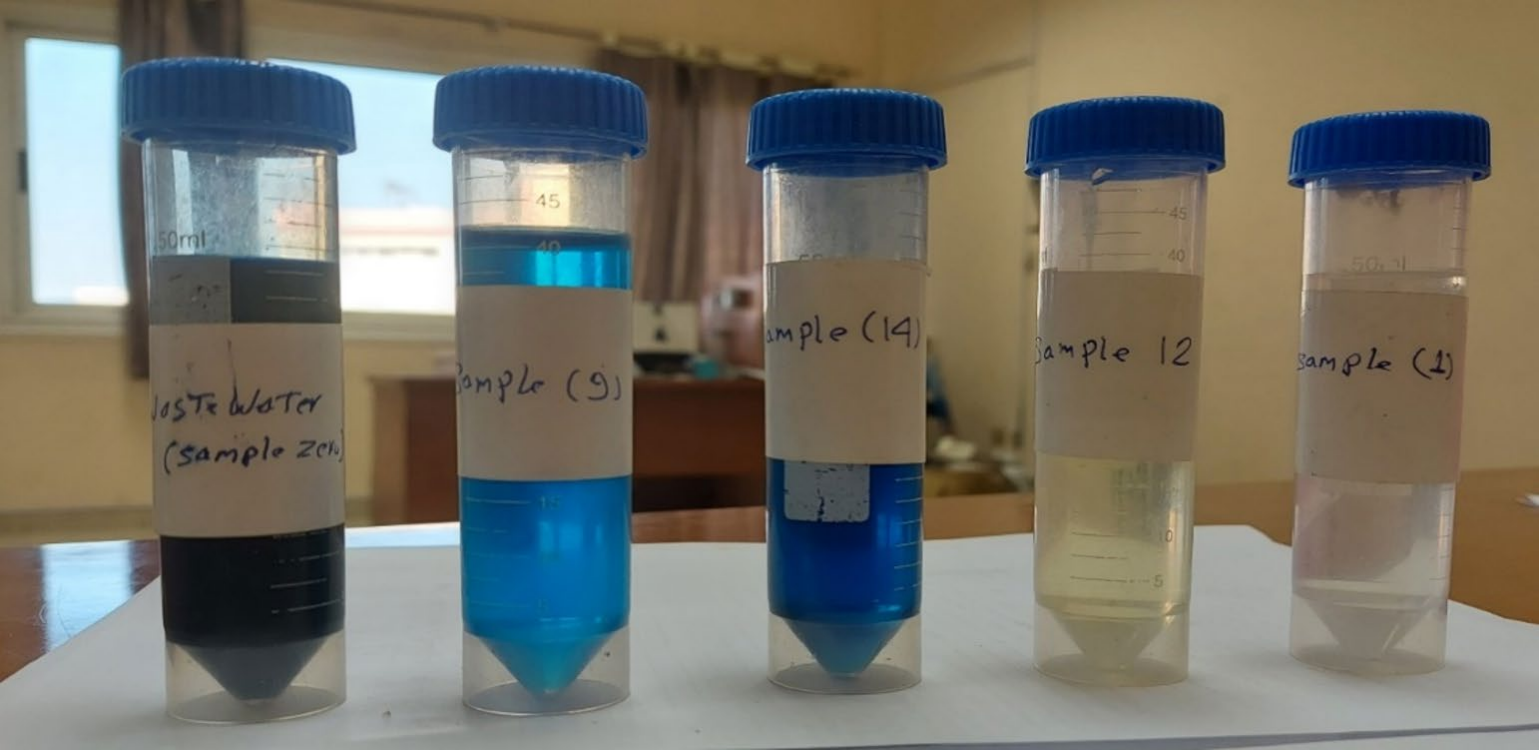
Photographs of samples treated under different conditions.

The catalyst dose “A” on the X-axis and hydrogen peroxide “B” on the Y-axis were studied while varying the pH.C. to its minimum, average, and maximum values to study its effect on dye removal. Figure 17a–c indicates that at a minimum pH, the removal efficiency increases as the peroxide dose increases; at the maximum pH, increasing the catalyst dose or peroxide dose increases the removal efficiency of the dye. Figure 18 shows photos of several treated samples after different runs.

**Figure 19:**
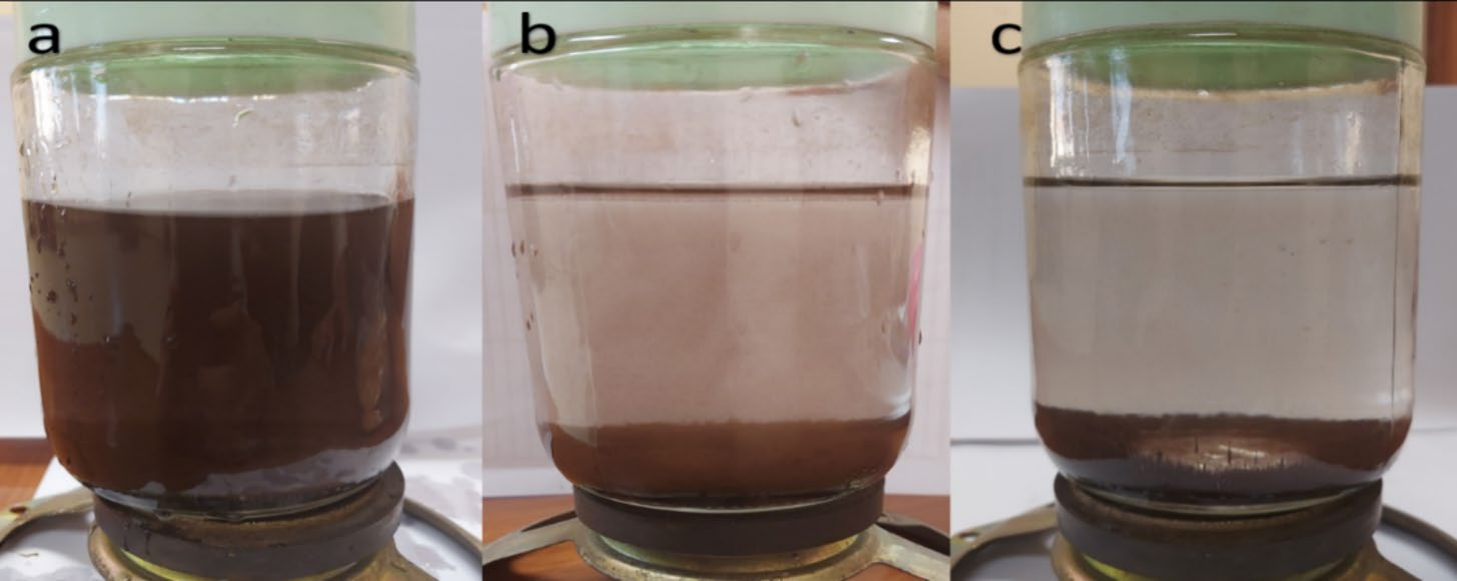
Separation of nanomagnetite from treated water using a magnet: (**a**) initial sample, (**b**) after 2 min, and (**c**) after 5 min.

**Figure 20:**
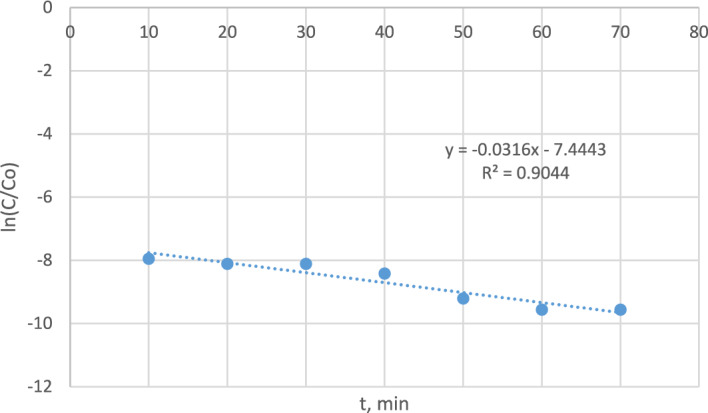
Kinetics of photocatalytic degradation of M.B dye using nanomagnetite.

Figure 19a–c shows the separation of magnetite nanoparticles from treated water using a magnet. The solution was very clear after approximately 5 min, as shown in Fig. 20c. All the magnetite nanoparticles were attracted to the magnet below the beaker, which confirmed the magnetic characteristics of the produced nanomagnetite and the ease of separation from treated water, which distinguishes it as a catalyst for advanced oxidation in wastewater treatment.

### Effect of UV radiation on the removal efficiency of methylene blue solution using advanced oxidation

Two samples of methylene blue dye (100 ppm) were prepared, and the same conditions were applied for treatment via advanced oxidation under the optimum conditions, as determined from the abovementioned study. One of the samples was subjected to UV radiation at different time intervals—10, 20, 30, 40, 50, 60, and 70 min—and the other sample was not. The concentration was measured for both samples at different times, and the removal efficiency was calculated as listed in Table 7. The removal efficiency was improved by using UV radiation and reached a maximum value of approximately 99% after 50 min.

**Table 7:** Effect of UV radiation on the oxidation process.

Time, min	Removal efficiency (%) (without UV radiation)	Removal efficiency (%) (with the application of UV radiation)
10	90	96.5
20	90	97
30	92	97
40	93	97.8
50	93.5	99
60	95	99.3
70	95	99.3

#### Kinetics of the photocatalytic oxidation of M.B dye using nanomagnetite

To investigate the photocatalytic degradation abilities of nanomagnetite, a quasifirst-order kinetic model was applied to analyze the kinetics of dye degradation, and the correlation equation is expressed as follows^34,35^:

16 $$ {\text{Ln}}\left( {{\text{C}}/{\text{Co}}} \right) = {\text{kt}} $$

C_o_ is the initial concentration of dye, C is the concentration of dye at any given time, and k is the rate constant. The linear relation between ln(C/C_o_) is represented by Fig. 20, and the rate constant k = 0.0316 min^−1^ is calculated from the slope of the line. The kinetic model is highly fitted to quasifirst-order kinetics, as R^2^ = 0.904.

#### Assessment of the reuse of synthesized magnetite nanoparticles

**Figure 21:**
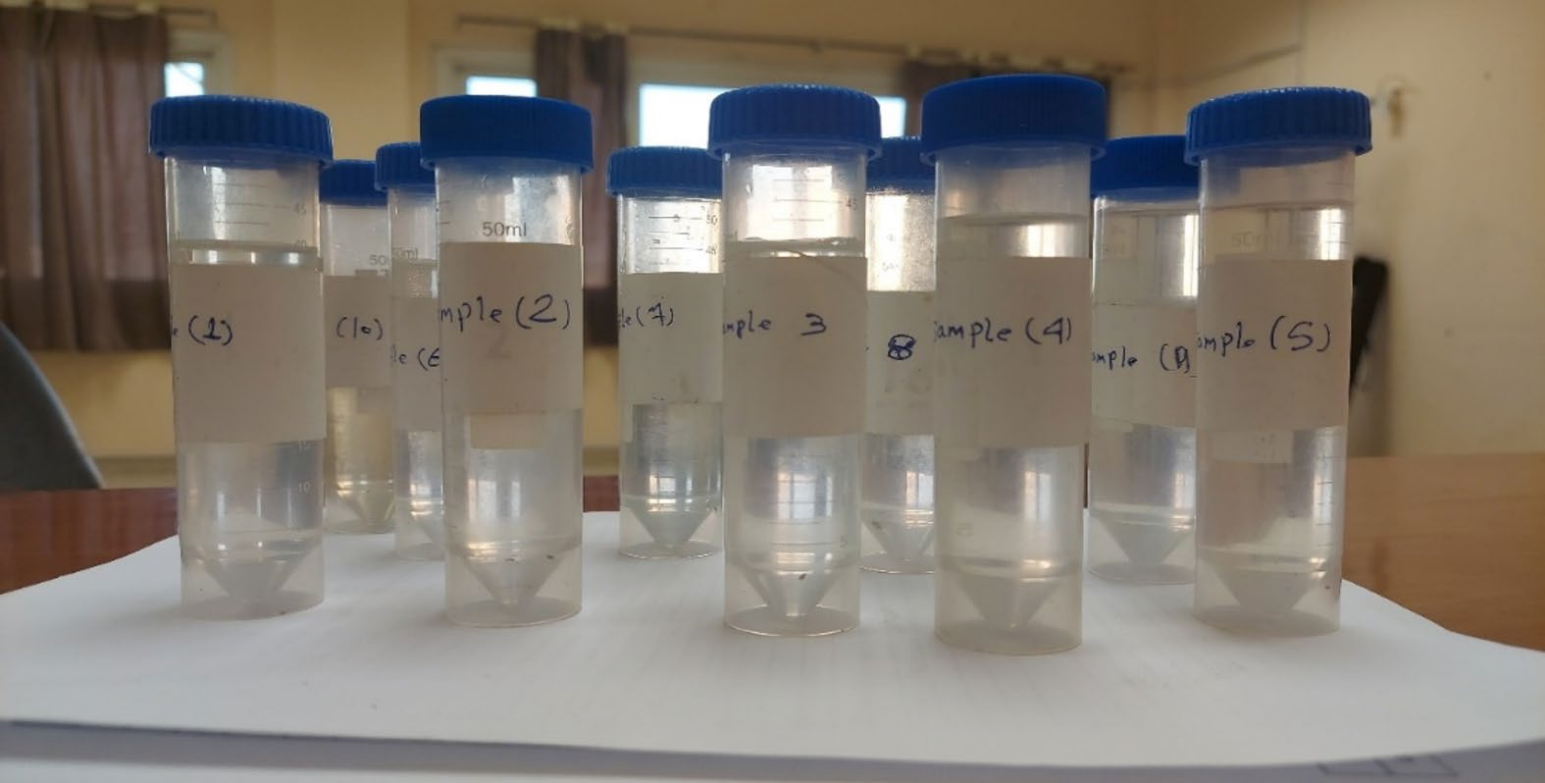
Magnetite reuse after 10 cycles.

A stock of 20 ppm methylene blue solution was prepared, and advanced oxidation was applied for the treatment of synthetic wastewater. Ten samples were prepared at the same concentration. The optimum conditions were applied after 24 h. The solution was withdrawn, and fresh synthetic wastewater was added to the same dose of magnetite. This process was repeated ten times to measure the efficiency of using reused magnetite as a catalyst. As shown in Table 8, the removal efficiency decreased from 100 to 90% after using magnetite ten times, which supports that the treatment process is economical, as shown in Fig. 21.

**Table 8:** Magnetite reuse results in terms of removal efficiency.

Cycle	Removal efficiency%
1	100
2	100
3	95.75
4	93.17
5	93.12
6	93.02
7	92
8	91.5
9	90.5
10	90

#### Industrial samples

Industrial waste samples were taken from the industrial zone at the tenth Ramadan textile factory, and several experiments were conducted. The initial and final COD concentrations were 1500 PPM and 195 PPM, respectively, which corresponds to 87.5% removal efficiency at this pH 2, 1250 peroxide dose and 200 ppm magnetite.

## Conclusion

Magnetite nanoparticles were prepared via the coprecipitation of ferrous and ferric oxides. The prepared nanomagnetite powder was characterized using XRD, which confirmed the formation of single-phase magnetite, and the zeta potential data indicated that the particles had a negative charge when dispersed in solution. TEM confirmed the formation of spherical nanoscale magnetite particles with an average size of 29.2 nm. The magnetic properties of the prepared sample were tested via VSM, which confirmed the attraction of the sample to a magnet with a magnetization of 60 (emu/g).

The prepared nanomagnetite powder was applied in wastewater contaminated with methylene blue dye using both adsorption and advanced oxidation techniques.

During the treatment of wastewater via adsorption, the highest removal efficiency (95.11%) was obtained by adding 50 mg of Fe_3_O_4_/L, 10 ppm dye conc, and a pH of 6.5. The adsorption results were fitted to the Langmuir model, which indicated that a monolayer was formed, and the Dubinin–Radushkevich isotherm had an activation energy of 2.23 kJ/mol, which indicated that the adsorption of M.B by nanomagnetite was a physisorption process. The adsorption kinetics and thermodynamics were studied, and the results showed that the pseudo first-order model was fitted with the experimental data, and the value of ΔH^0^ was positive, which indicated an endothermic reaction. The value of ΔS^0^ was positive, which indicated that the degrees of freedom increased at the solid–liquid interface during dye adsorption.

An advanced oxidation process using peroxide and magnetite nanoparticles was applied for the treatment of methylene blue dye solution (100 ppm). RSM was applied using different parameters: pH 2:11, 20:200 mg Fe_3_O_4_/L and 500:2000 ppm of peroxide. The highest removal efficiency (98.5%) was obtained by adding 110 PPM magnetite to 2000 ppm H_2_O_2_ at a pH = 11. The kinetic model was studied and found to be highly fit to quasifirst-order kinetics, with R^2^ = 0.904.

COD was measured for various samples, and the removal efficiency of the contaminated organic matter was 92.2% for the measured optimum sample. The magnetite was reused for ten cycles under optimum conditions, and the removal efficiency was reduced by 10%.

## Data Availability

Data is provided within the main manuscript file.
